# Supplementary material for: Differential Effect of Extracellular Acidic Environment on IL-1β Released from Human and Mouse Phagocytes
Source: Int J Mol Sci. 2020 Sep 30;21(19):7229. doi: 10.3390/ijms21197229 (PMC7582253; doi:10.3390/ijms21197229)

Supplementary Figures

Differential effect of extracellular acidic environment on IL-1β released from human and mouse phagocytes

Petra Sušjan, Mojca Benčina, Iva Hafner-Bratkovič

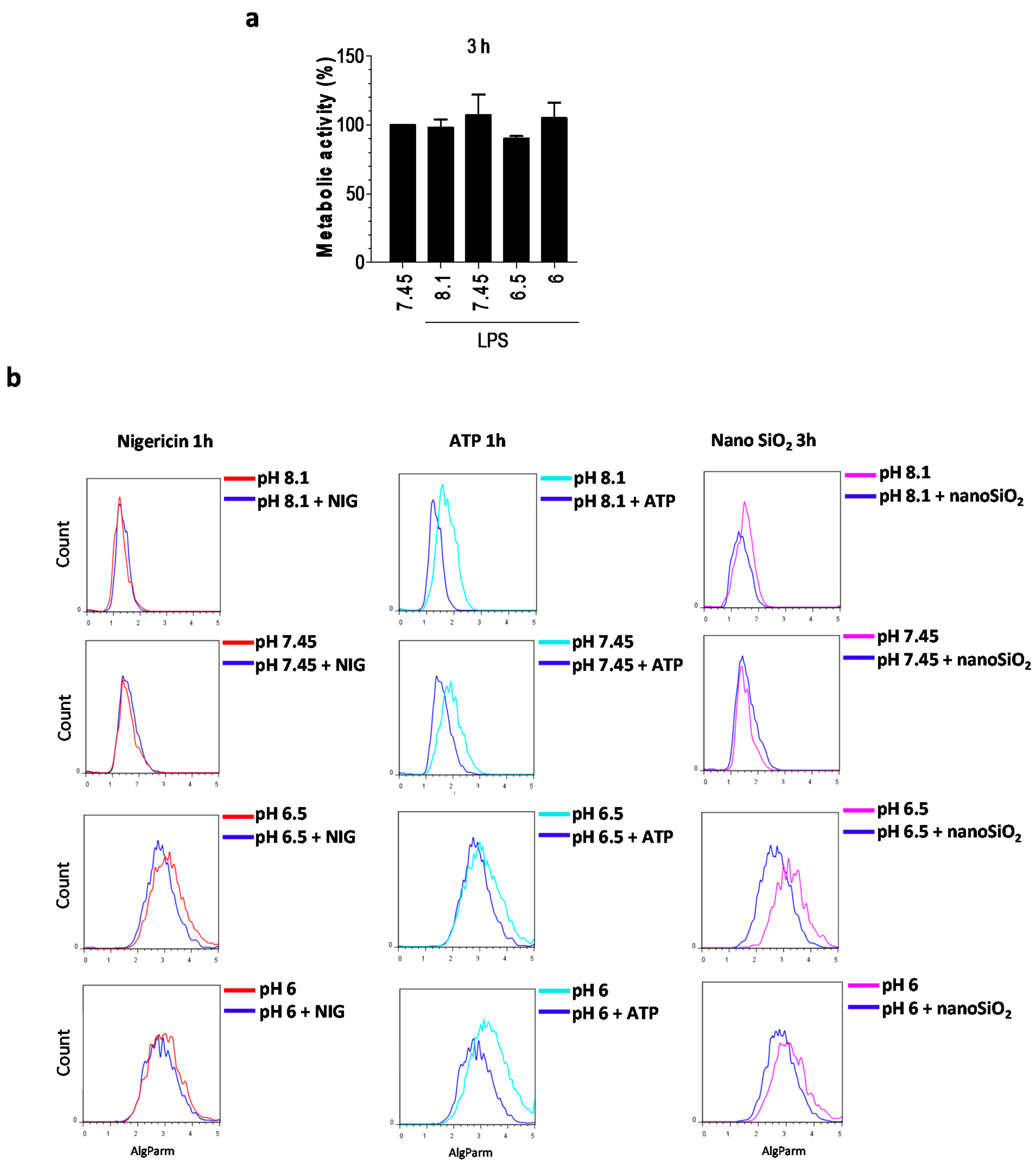

Supplementary Figure 1. Cell viability and estimation of the intracellular pH shift by genetically encoded sensor. (a) Metabolic activity of cells by XTT assay after iBMDMs were primed with 100 ng/mL LPS for 6 h and exposed to buffers with various pH for 3 h. **(b)** Count of cells with deprotonated or protonated pHluorin with ratiometric cytometry after iBMDMs constitutively expressing pHluorin were primed with 100 ng/mL LPS for 6 h and then treated with 10 µM nigericin, 5 mM ATP or 180 ng/mL nanoSiO_2 in buffers with various pH. Subsequently cells were detached and ratiometric cytometry method was used to determine the ratio between fluorescence at 488 nm and 405 nm (AlgParm).

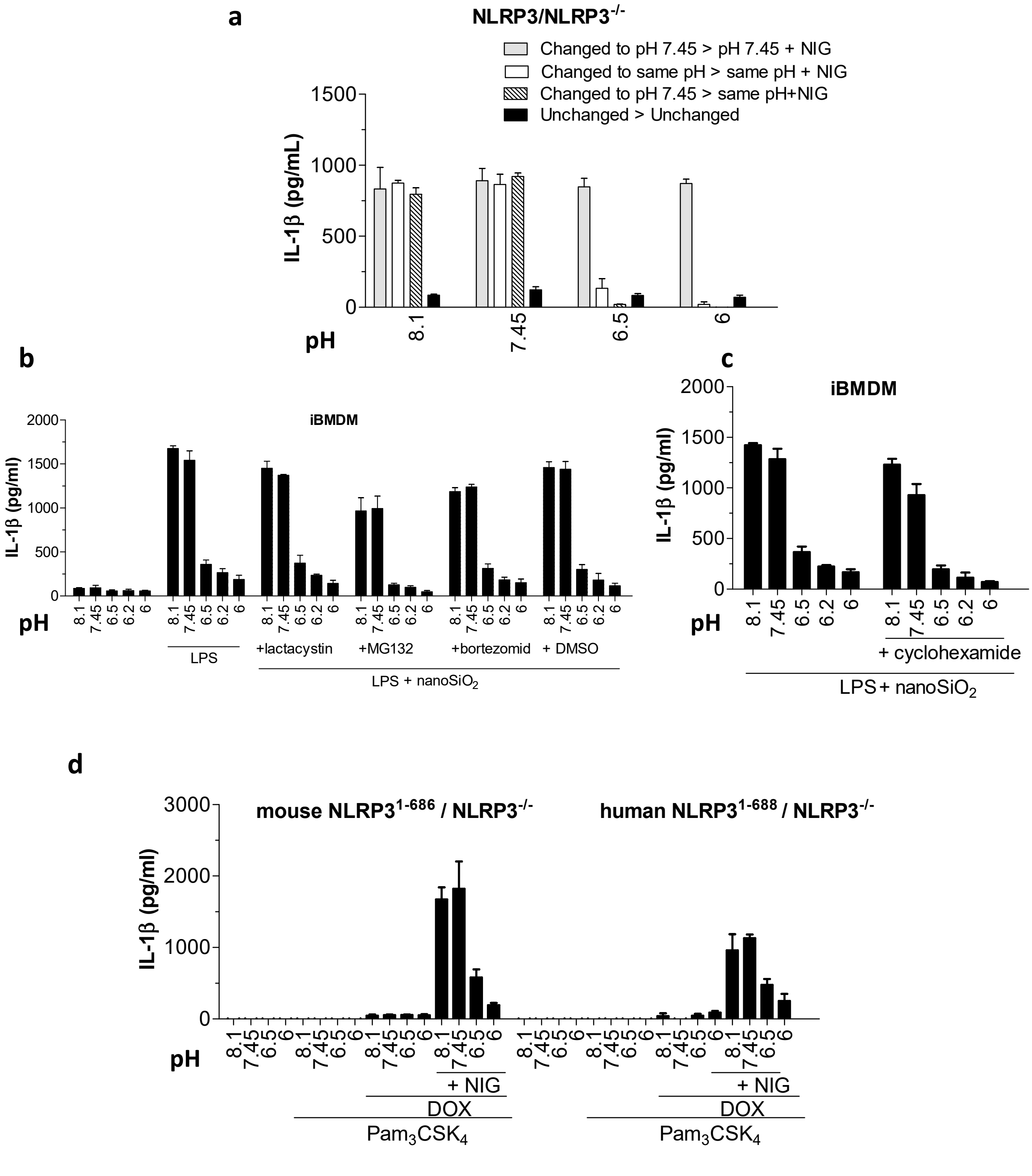

Supplementary Figure 2. No role of proteasome degradation in pH-mediated inflammasome suppression. (a) IL-1β levels by ELISA after iBMDMs were primed with 100 ng/mL LPS for 6 h and then incubated for 1h in buffers with various pH. In the next hour cells were either kept in same pH (unchanged), replaced with a buffer pH 7.45 or replaced by the same pH buffer. In the third hour, buffers either again remained unchanged, replaced with a buffer pH 7.45 containing 10 μM nigericin or replaced by the same pH buffer containing nigericin. **(b,c)** IL-1β levels by ELISA released from iBMDMs after priming as in (a) and 3h nanoSiO2 (180 μg/mL) stimulation in the

presence of proteasome inhibitors lactacystin (50 µM), MG132 (20 µM) and bortezomid (10 µM) (b) or translation inhibitor cycloheximide (100 µM) (c). **(d)** IL-1β levels by ELISA released from NLRP3$^{-/-}$ iBMDMs expressing mouse NLRP3$^{1-686}$ or human NLRP3$^{1-688}$ after overnight priming with 200 ng/mL Pam_3CSK_4 and 0.5 µg/mL doxycycline, followed by 1 h incubation in buffers with various pH alone or containing 10 µM nigericin.

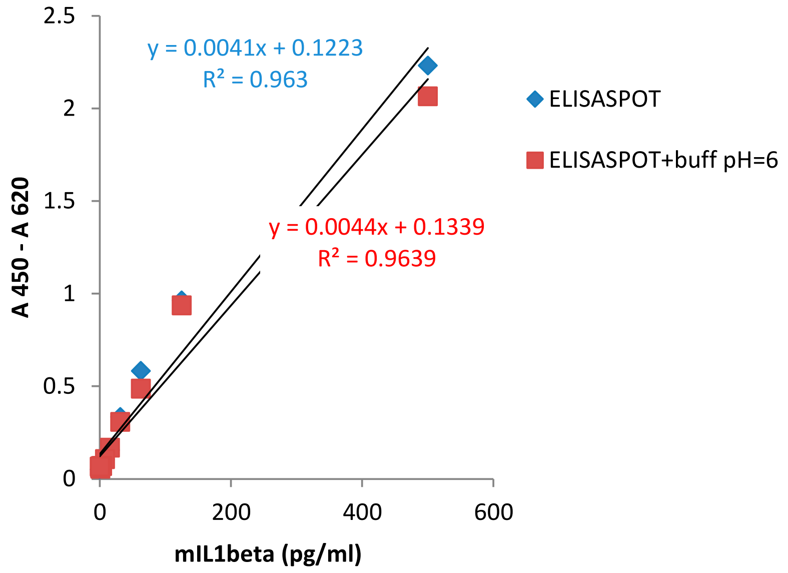

Supplementary Figure 3. The functionality of ELISA test in various pH conditions. Mouse IL-1β standard from IL-1 beta uncoated ELISA Kit was diluted in either ELISASPOT block solution or in a 1:1 mixture of blocking solution and buffer with pH 6 as normally done when testing samples. We then performed ELISA and compared the standard trend lines with R^2 efficiency coefficient.

Uncropped Western blots:

Figure 2f, β-actin

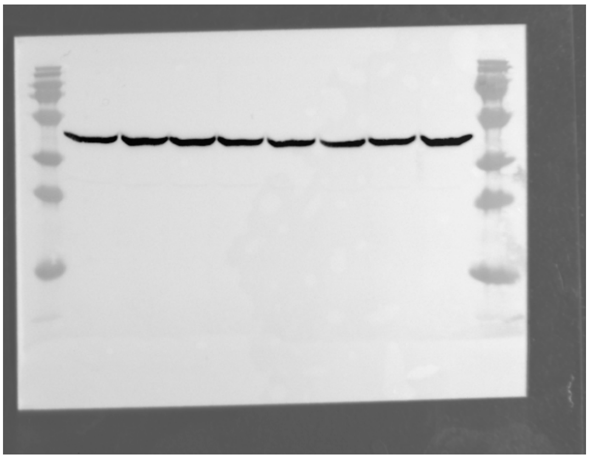

Figure 2f, proIL1beta (in red) and IL1beta p18 (in blue)

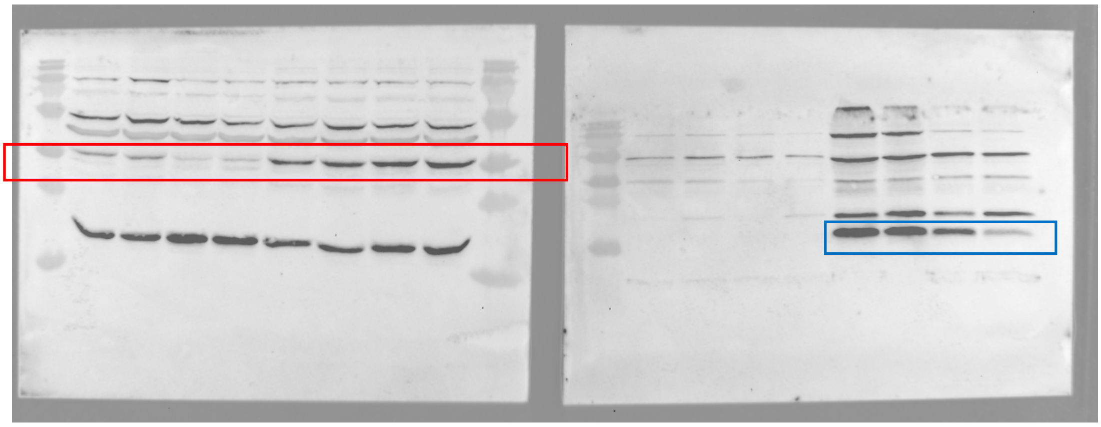

Figure 4a, β-actin (in red), ASC (in blue). Images represent same blot but in different exposition times.

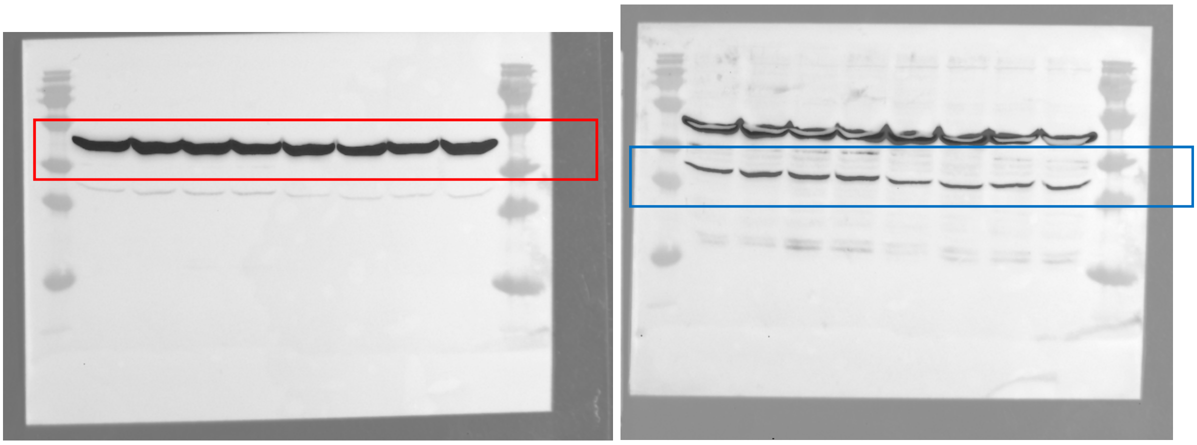

Figure 4a, NLRP3 (red), β-actin (blue)

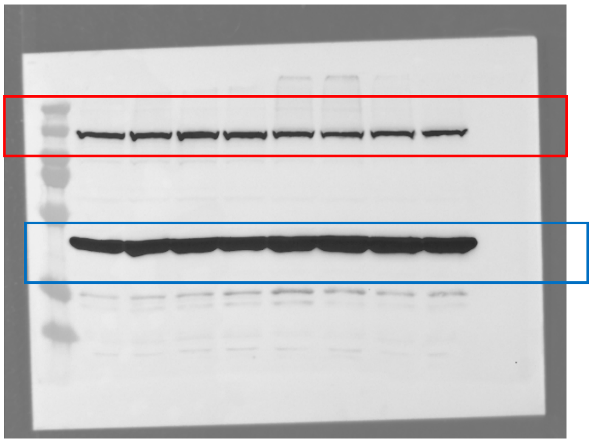

Figure 4a, Procaspase-1 (red), caspase-1 p20 (blue)

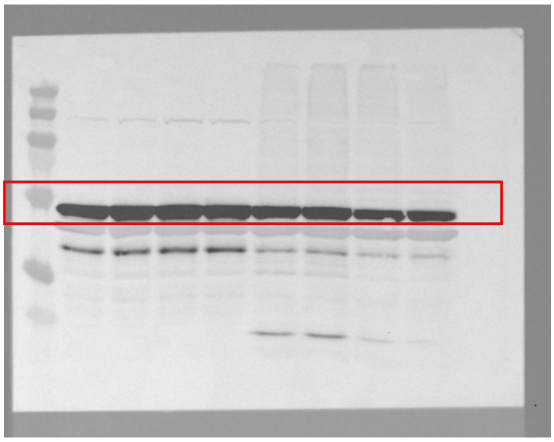

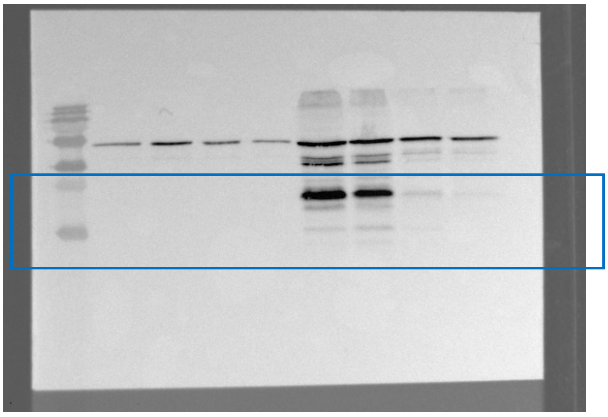

Supplement: Supplementary file 1 [file ijms-21-07229-s001.pdf]
